# Elevated levels of fibrinogen-derived endogenous citrullinated peptides in synovial fluid of rheumatoid arthritis patients

**DOI:** 10.1186/ar3840

**Published:** 2012-05-14

**Authors:** Reinout Raijmakers, Joyce JBC van Beers, Mahmoud El-Azzouny, Natasja FC Visser, Borut Božič, Ger JM Pruijn, Albert JR Heck

**Affiliations:** 1 Biomolecular Mass Spectrometry and Proteomics Group, Bijvoet Center for Biomolecular Research and Utrecht Institute for Pharmaceutical Sciences, Utrecht University and Netherlands Proteomics Centre, Padualaan 8, Utrecht, 3584 CH, The Netherlands; 2 Department of Biomolecular Chemistry, Nijmegen Center for Molecular Life Sciences, Institute for Molecules and Materials, Radboud University Nijmegen, Geert Grooteplein 26, Nijmegen, 6525 GA, The Netherlands; 3 University Medical Centre Ljubljana, Department of Rheumatology, Immunology Laboratory, Vodnikova 62, Ljubljana, 1000, Slovenia

## Abstract

**Introduction:**

Rheumatoid arthritis (RA) is an autoimmune disease characterized by inflammation of the joints and the presence of autoantibodies directed against proteins containing the non-standard arginine-derived amino acid citrulline. The protein fibrinogen, which has an essential role in blood clotting, is one of the most prominent citrullinated autoantigens in RA, particularly because it can be found in the inflamed tissue of affected joints. Here, we set out to analyze the presence of citrullinated endogenous peptides in the synovial fluid of RA and arthritic control patients.

**Methods:**

Endogenous peptides were isolated from the synovial fluid of RA patients and controls by filtration and solid phase extraction. The peptides were identified and quantified using high-resolution liquid chromatography-mass spectrometry.

**Results:**

Our data reveal that the synovial fluid of RA patients contains soluble endogenous peptides, derived from fibrinogen, containing significant amounts of citrulline residues and, in some cases, also phosphorylated serine. Several citrullinated peptides are found to be more abundantly present in the synovial fluid of RA patients compared to patients suffering from other inflammatory diseases affecting the joints.

**Conclusions:**

The increased presence of citrullinated peptides in RA patients points toward a possible specific role of these peptides in the immune response at the basis of the recognition of citrullinated peptides and proteins by RA patient autoantibodies.

## Introduction

Rheumatoid arthritis (RA) is one of the most common autoimmune diseases, for which many autoantigens have been identified over the last decades. The most remarkable but yet most common targets of autoantibodies in RA are proteins containing the amino acid citrulline. This group of proteins includes, among others, histones and fibrin(ogen) [1-3]. Autoantibodies against citrullinated proteins (ACPA) are almost exclusively present in RA and can be present years before disease onset [4]. Detection of ACPA or other autoantibodies is a common part of the clinical diagnosis of RA. ACPA are often detected using the so-called cyclic citrullinated peptide, or CCP test [5].

Fibrinogen is a heteromultimeric protein consisting of two copies each of three different peptide chains (alpha, beta and gamma) [6]. It plays an essential role in the clotting cascade, where it is proteolytically cleaved by thrombin to form the clotting protein fibrin [7]. Citrullinated forms of the alpha and beta chains of fibrinogen are known to be present in the serum and synovial fluid (SF) of RA patients and can be targets for ACPA [1,8]. The role of citrullinated proteins and antibodies recognizing these proteins in the pathophysiology of RA is not fully understood, but specific human leukocyte antigen (HLA) alleles (HLA-DR4 and HLA-DR1, HLA-DR10 and HLA-DR14) have been linked to a predisposition to develop RA and the associated major histocompatibility complex (MHC) molecules were found to show increased ability to present peptides containing a citrulline residue [9,10]. Importantly, HLA-DR4 transgenic mice are susceptible to develop arthritis when immunized with citrullinated fibrinogen [8]. Recently, Ho and others found that mice that were immunized with citrullinated fibrinogen developed arthritis and fibrinogen-reactive T cells which produce the proinflammatory cytokines IL-6, IL-17, TNF-alpha, and IFN-gamma and that these mice possess rheumatoid factor (RF), circulating immune complexes and anti-CCP antibodies, all of which are characteristics of human RA [11]. Inhibition of the enzymes responsible for the generation of citrulline residues in proteins, the peptidylarginine deiminases (PADs), has been shown to reduce symptoms in mouse models of RA [12]. Immune complexes consisting of ACPA and citrullinated fibrinogen can also be found in a large part of the ACPA-positive RA patients [13] and are known to induce macrophage secretion of TNF-alpha, an important mediator of inflammation in RA [14].

Although the presence of citrullinated fibrinogen protein has attracted the most attention lately, it is known that fibrinogen-derived peptides can be present in the synovial fluid of inflamed joints [15] and that (citrullinated) peptides can be potent activators of cells of the immune system [8,16]. Here, we analyzed the synovial fluid obtained from 11 RA patients and 10 patients with related inflammatory diseases of the joint for the presence of endogenous soluble citrullinated peptides by liquid chromatography-tandem mass spectrometry (LC-MS/MS). We found that fibrinogen-derived citrullinated peptides were significantly more abundant in the fluid of the RA patients compared to the control patients, while their noncitrullinated counterparts were present at similar levels.

## Materials and methods

### Synovial fluid samples

Synovial fluid samples were obtained from 11 RA patients and 10 patients with other inflammatory diseases (four with psoriatic arthritis, two with ankylosing spondylitis, two with gout, one with osteoarthritis and one with ochronosis). Synovial fluid was obtained by joint punctures (arthrocentesis), using needles with a diameter from 1.6 to 2.2 mm. A sample was immediately centrifuged at 2,500 × g for 10 minutes at 4°C. Supernatant and pellet were separately stored at -80°C. All procedures were performed within two hours. All samples were collected for diagnostic purposes and all patients gave informed consent that the samples could also be used anonymously for research purposes, in accordance with the ethical guidelines as established by the ethics committee of the University Medical Centre Ljubljana (Slovenia).

### Isolation of peptides from synovial fluid

Peptides were isolated from synovial fluid based on the protocol recently described by Kamphorst *et al*. [15]. First, intact proteins were removed from the synovial fluid by ultracentrifugation. Spin filter columns with a 10 kD cutoff were first washed with 100 μl ethanol by centrifugation for 10 minutes at 11,000 × g at 4°C and then with 100 μl 50 mM NH_4_HCO_3 _pH 7, also for 10 minutes at 11,000 × g at 4°C. Next, 50 μl of synovial fluid was mixed with 235 ul of 50 mM NH_4_HCO_3 _pH 7 and 15 μl DMSO and was passed through the spin filter by centrifugation for 60 minutes at 11,000 × g at 4°C. The spin filter was then washed again with 100 μl 50 mM NH_4_HCO_3 _pH 7 for 10 minutes at 11,000 × g at 4°C and the filtrates of the last two steps were combined and acidified with 5 μL formic acid (FA). Next, Sep-Pak C18 (Waters Corp., Milford, MA, USA) disposable cartridges with a capacity of 50 mg were activated with 1 ml acetonitrile (ACN) and conditioned with 1 ml 2.5% ACN/1% FA. After passing the filtrates over the cartridges, they were washed with 1 ml 2.5% ACN/1% FA. Finally, peptides were eluted with 150 μL 80% ACN/1% FA and dried by vacuum centrifugation. For mass spectrometry (MS) analysis, dried peptide samples were reconstituted in 50 μl of 5% FA. Five μl of each sample was spiked with 1 μl of internal standard, containing three standard peptides with mass-to-charge ratio (m/z) of 556.28, 477.78, and 497.60.

### Identification and quantitation of endogenous peptides by LC-MS/MS

Isolated synovial fluid peptides were analyzed by nanoscale liquid C18 chromatography using an Agilent 1100 series LC system coupled to an LTQ-FT-ICR mass spectrometer (Thermo Fisher Scientific Inc., Waltham, MA, USA). The mass spectrometer was operated in the data-dependent mode to automatically switch between MS and MS/MS acquisition and the five most intense ions were fragmented in the linear ion trap using collisionally induced dissociation.

Obtained MS/MS spectra were analyzed by database searching against all human proteins in the SwissProt database using the Mascot software platform version 2.1.0 (Matrix Science Ltd., London, UK). The search criteria included methionine oxidation, arginine citrullination and asparagine and glutamine deamidation as variable modifications. The peptide tolerance was set to 5 ppm and the MS/MS tolerance to 0.6 Da. All searches were done without enzyme restrictions and peptides were, unless described differently, identified with a minimum Mascot score of 35. For spectral counting, peptide spectrum matches with a Mascot score of at least 20 were included. Technical details of the LC-MS/MS analysis and database searching can be found in Additional file 1.

## Results

To assess the presence of citrullinated endogenous peptides in the synovial fluid of RA patients, synovial fluid and serum was collected from 11 RA patients and 10 patients with other inflammatory diseases of the joint. The ACPA (anti-citrullinated protein antibody) status of all RA patients was assessed by ELISA using the CCP2 test and is given in Additional file 1, Table S1. Endogenous peptides with a size smaller than 10 kDa were isolated from the synovial fluid by ultrafiltration and solid phase extraction.

### Identification of endogenous citrullinated peptides

**Figure 1:**
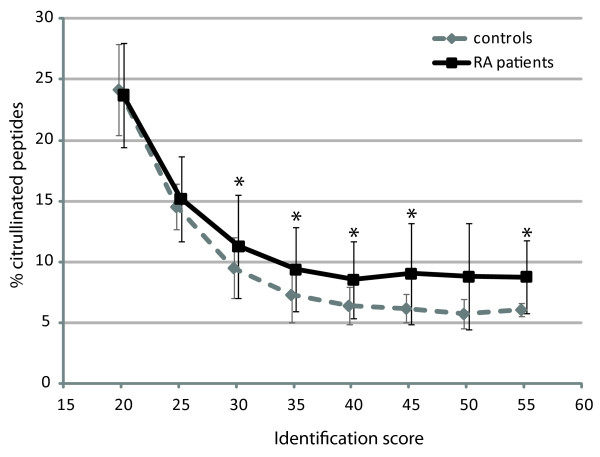
**Identified citrullinated peptides at various identification scores**. Graph showing the average percentage of all identified peptides that contain at least one citrulline residue at varying identification threshold scores in the synovial fluid of rheumatoid arthritis (RA) patients (black line) and control patients (dashed grey line). Bars indicate the median absolute deviations and asterisks indicate the scores at which a statistically significant difference (*P *< 0.05) was observed, as determined by a Mann-Whitney test.

**Figure 2:**
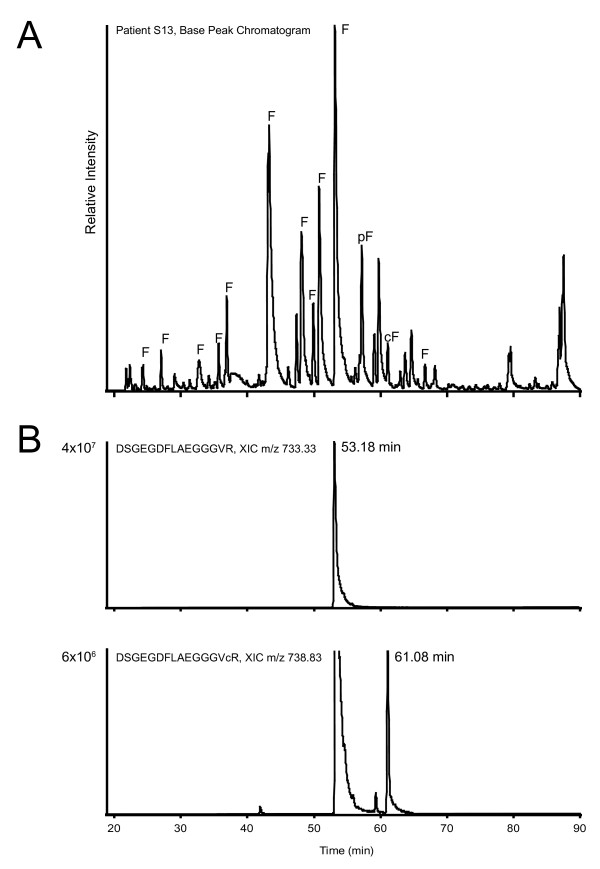
**Chromatography of synovial fluid and fibrinogen-derived peptides**. Panel A contains a base peak chromatogram of the LC-MS/MS analysis of the peptides in the synovial fluid of rheumatoid arthritis (RA) patient S13, showing the complexity of the sample. All base peaks derived from the alpha chain of fibrinogen are indicated with 'F', with citrullinated and phosphorylated peptides indicated with 'cF' and 'pF', respectively. Panel B shows the extracted ion chromatograms (XIC) of peptide DSGEGDFLAEGGGVR (mass-to-charge ratio (m/z) 733.33), which elutes at a retention time (RT) of 53.18 minutes and the citrullinated version of the same peptide (m/z 733.83), eluting at 61.08 minutes. Note that in the bottom chromatogram also a peak is visible at the elution time of the noncitrullinated peptide, corresponding to the ^13^C isotopic peak of that peptide, which has a mass very close to the citrullinated peptide and is, therefore, also extracted by the algorithm. The relative areas of the two extracted chromatograms are indicated at the y-axis of the XICs. LC-MS/MS, liquid chromatography-tandem mass spectrometry.

The synovial fluid-derived peptides were analyzed by LC-MS/MS on a high-resolution LTQ-FT-ICR mass spectrometer and the resulting spectra were searched against all human proteins in the SwissProt database, allowing for citrullination, deamidation and oxidation as variable modifications. Because citrullination leads to an additional mass of nominally one Dalton compared to regular arginine residues, such searches are prone to false positives stemming from the misidentification of ^13^C isotopes or modifications that lead to similar nominal mass increases (for example, deamidation of asparagines or glutamine). Such false, randomly matched, identifications are expected to have lower identification scores, because the identification score itself is calculated using the probability that an observed match between experimental data in the MS/MS spectra and a database sequence is random. We plotted the average relative number of peptides containing citrulline residues at various cutoff scores for patient and control samples (Figure 1). This showed that, at lower scores, the percentage of identified citrullinated peptides was almost 25% in patient samples and that this value dropped when higher cutoff scores were used leveling off at approximately 10% at cutoff scores of 35 and higher. Therefore, we chose to include only peptides identified with a score of at least 35 for further analysis. This analysis also showed that, irrespective of score cutoff, the average number of citrullinated peptides identified in RA patients was higher than in control patients. Because we were mainly interested in citrullinated peptides elevated in the RA patients, we generated a first list of candidate peptides based on their spectral counts in the two patient groups. We selected all peptides that were identified in at least two more RA patients than control patients and had an overall spectral count which was 2.5 times higher in the RA patients compared to controls. This resulted in a list of 17 citrullinated peptides, 15 of which we identified as being derived from the alpha chain of fibrinogen (Table 1). This is similar to previous reports [15] and is not unexpected, as (citrullinated) fibrinogen alpha is known to be highly abundant in the synovial fluid proteome [3] and, unlike many other proteins, undergoes efficient proteolysis as part of the blood clotting cascade. While other proteins might be even more abundantly present in the synovial fluid (for example, albumin or antibodies) [17], these would not be detected in this study, because they are not as commonly proteolytically processed as fibrinogen. Figure 2A shows the LC-MS chromatogram of the synovial fluid of patient S13, with all major peaks corresponding to fibrinogen-derived peptides indicated to illustrate that these peptides dominate the chromatogram.

**Table 1:** List of citrullinated peptides identified more often in rheumatoid arthritis patients compared to control patients.

Sequence	Protein	Accession	Score
**DSGEGDFLAEGGGVcR**	Fibrinogen α chain	FIBA_HUMAN	87.8

**SGEGDFLAEGGGVcR**	Fibrinogen α chain	FIBA_HUMAN	67.8

**EGDFLAEGGGVcR**	Fibrinogen α chain	FIBA_HUMAN	73.2

**FLAEGGGVcR**	Fibrinogen α chain	FIBA_HUMAN	50.9

**EEVSGNVSPGTRcR**	Fibrinogen α chain	FIBA_HUMAN	51.5

**EEVSGNVSPGTRcRE**	Fibrinogen α chain	FIBA_HUMAN	69.8

**EEVSGNVSPGTcRREY**	Fibrinogen α chain	FIBA_HUMAN	60.0

**EEVSGNVSPGTRcREY**	Fibrinogen α chain	FIBA_HUMAN	48.8

**ELEcRPGGNEITRGGSTSY**	Fibrinogen α chain	FIBA_HUMAN	50.1

**NcRGDSTFESKSY**	Fibrinogen α chain	FIBA_HUMAN	69.0

**DEAGSEADHEGTHSTKcRGHA**	Fibrinogen α chain	FIBA_HUMAN	40.8

**GSTGNcRNPGSSGTGGTATWKPGSSGP**	Fibrinogen α chain	FIBA_HUMAN	59.1

**SETEScRGSESGIFTNTK**	Fibrinogen α chain	FIBA_HUMAN	49.6

**SSSYSKQFTSSTSYNcRGDSTF**	Fibrinogen α chain	FIBA_HUMAN	41.9

**TcRGGSTSYGTGSETESPRNPS**	Fibrinogen α chain	FIBA_HUMAN	52.0

**TSEASSSSSSSSSSSRScRScRSL**	Peroxisome proliferator-activated receptor gamma coactivator-related protein 1	PPRC1_HUM	68.1

**GLADNTNDLEKcRcR**	Plasma membrane calcium-transporting ATPase 3	AT2B3_HUMAN	40.5

Note: cR indicates the position of a citrulline residue.

**Figure 3:**
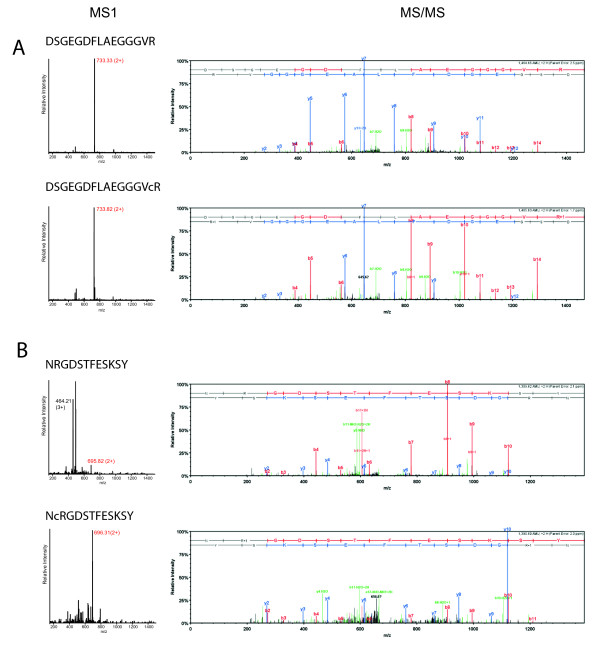
**Identification of citrullinated peptides**. MS1 (left) and MS/MS (right) spectra of two different fibrinogen-derived peptides (DSGEGDFLAEGGGVR in panel A and NRGDSTFESKSY in panel B) from synovial fluid samples. In the MS1 spectra, the peak(s) corresponding to the respective peptide are marked with their mass-to-charge ratio (m/z) and charge state, the peak of which the MS/MS spectrum is shown and is marked with the red m/z value. In the corresponding MS/MS spectra, y-ions are marked in blue, b-ions are marked in red and all other annotated ions are marked in green. Please note that parent ions are not present in the MS/MS spectra generated with the instrumentation and settings used. MS, mass spectrometry; MS/MS, tandem mass spectrometry.

Given the difficulties with the reliable identification of citrullinated peptides described above, further analyses were performed to unambiguously confirm the citrullination of each of these 17 peptides. Misidentification of ^13^C isotopes was prevented by choosing a stringent precursor tolerance (5 ppm) for the initial searches, taking into account that ^13^C leads to a larger mass difference (1.0036 Da) than citrullination (0.9802 Da). Deamidation, on the other hand, cannot be discriminated from citrullination based upon mass alone. However, deamidation does not change the number of charged residues in the peptide (under the acidic conditions used for LC-MS), whereas the conversion of arginine to citrulline neutralizes a positive charge. Therefore, we anticipated citrullination to have a pronounced effect on ionization and fragmentation of the peptide, and on the elution time of the peptide in the reversed phase separation preceding our MS analysis. We analyzed the retention times of all peptides in Table 1 and compared them with the corresponding noncitrullinated peptides. This revealed that, on average, citrullinated peptides eluted five minutes later from the C18 column than their arginine-containing counterparts (Figure 2B and Additional file 1, Table S2), in agreement with their increased hydrophobicity. We also analyzed the precursor mass spectra and fragmentation spectra for all of these peptides and found that citrullination caused the dominant precursor charge state to be lower for peptides with a charge of three and higher (Figure 3 and Additional file 1, Table S2), whereas deamidation had no such effect (data not shown). Also the MS/MS spectra changed upon citrullination, as anticipated, with more prominent b-ions showing up when the converted arginine was on the C-terminal part of the peptide and increased levels of y-ions when it was located more N-terminally (Figure 3).

### Quantification of endogenous citrullinated peptides

**Figure 4:**
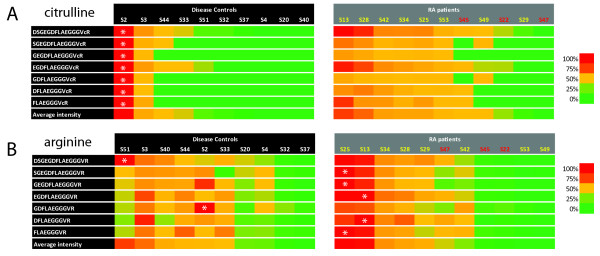
**Abundance of fibrinopeptide A-derived peptides in rheumatoid arthritis (RA) patients and controls**. Heat map displaying the relative abundance as determined from the extracted ion chromatograms, (XIC) of citrullinated peptides (panel A) and their noncitrullinated counterparts (panel B) in RA patients and control patients. Because the abundance of different peptides cannot be directly compared due to their different behavior in the mass spectrometer, the colour coding is applied per peptide sequence, with the patient containing the highest amount of each particular peptide coloured in red, the lowest coloured in green and the other patients coloured according to the colour scale shown on the right of the panel. White asterisks indicate in which patient the highest abundance of a specific peptide was observed. For the RA patients, the code of CCP2+ patients is shown in yellow and of CCP2- patients in red. Patients are sorted according to the average abundance of all listed peptides, shown at the bottom of each panel. CCP, cyclic citrullinated peptide.

Having robustly established the citrullinated identity of these peptides, we set out to analyze their abundance in the synovial fluid. From the sequence of the peptides, it was apparent that mostly two regions of fibrinogen were identified with multiple peptides, with different termini, probably due to endogenous proteolytic activity. These two regions span amino acids 20-35 (sequence ADSGEGDFLAEGGGVR) and 414-433 (sequence EEVSGNVSPGTRREYHTEKL) of the fibrinogen alpha chain. The first sequence corresponds to the so-called fibrinopeptide A, a signal peptide generated from the N-terminus of fibrinogen alpha during blood clotting. Because of their prominent presence, we decided to focus on these regions, and to include other peptides covering these sequences in further analyses. We determined the relative abundance of all selected peptides in all samples by calculating the area of their extracted ion chromatograms (XIC) in the LC-MS analyses (as is shown for peptide DSGEGDFLAEGGGVR in Figure 2B), normalized using spiked (citrullinated) synthetic peptides. Figure 4A shows a heat map displaying the abundance of each of these citrullinated peptides for fibrinogen region 20-35 in the synovial fluid samples, suggesting that some of them are more often present in high amounts in RA patients compared to controls. This effect can be attributed both to an increase in the total amount of peptide present and to an increased level of citrullination. Therefore, we also calculated the extract ion chromatogram areas for all corresponding noncitrullinated peptides (Figure 4B). This revealed that the increased levels observed for the citrullinated peptides were not seen for their noncitrullinated counterparts, suggesting an increased level of citrullination. A similar analysis of region 414-433 is shown in Additional file 1, Figure S1 and showed a higher abundance of both the citrullinated and the noncitrullinated peptides in the RA patients.

**Figure 5:**
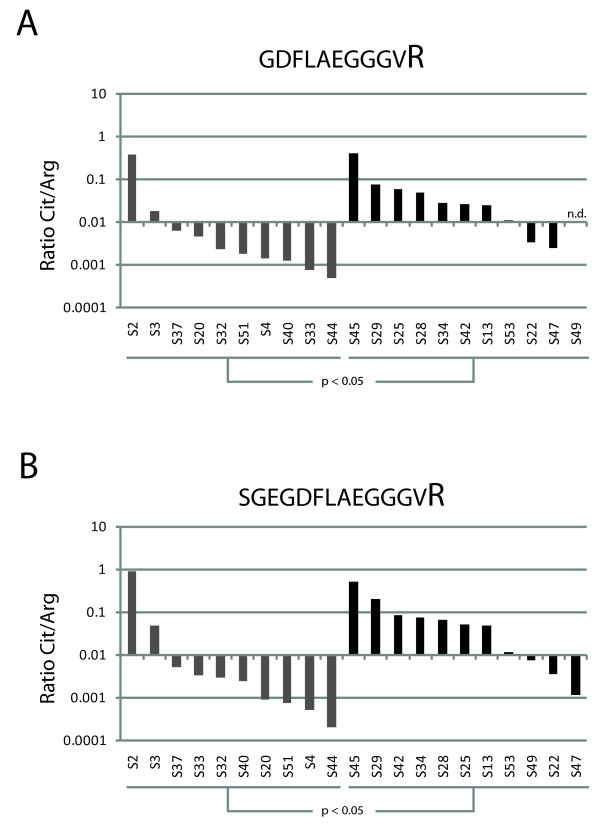
**Relative abundance of citrullinated peptides and their noncitrullinated counterparts**. Bar graphs showing the ratio between the abundance of a specific citrullinated peptide versus its noncitrullinated variant in rheumatoid arthritis (RA) patients (black bars) and control patients (gray bars) on a base 10 logarithmic scale. Panel A shows the ratios for peptide GDFLAEGGGVR and panel B for peptide SGEGDFLAEGGGVR. When no signal for the peptide was detected, a value equivalent to the background signal was assigned for this calculation. If both the signal of the citrullinated and the noncitrullinated peptide were missing, the ratio is shown as not determined (n.d.). Patients are sorted per group according to the observed ratios.

To visualize the changed levels of citrullination for the peptides covering amino acids 20-35 of fibrinogen alpha, we also calculated the average relative amount of selected citrullinated peptides versus their noncitrullinated counterpart by calculating the ratio between the observed areas for these peptides. This revealed that the noncitrullinated peptides are detected in roughly 100-fold higher amounts (shown in Figure 5 by the approximate average ratio of 0.01), but this does not reflect actual abundances because the arginine-containing peptides will be detected much more efficiently due to their higher basicity and thus ionization efficiency [18]. Still, the data can be interpreted relative to each other and although the difference between individual patients is large, it is clear that a significant increase of the citrullinated variants of the peptide was observed in the RA patients (Figure 5). For both combinations of corresponding citrullinated and noncitrullinated peptide sequences shown in Figure 5, a *t *test on the log-transformed areas revealed significant differences between RA patients and control patients with *P *values of < 0.05. In addition, for both sets also the categorical Fisher's exact test (using cutoff 0.01) confirmed the significant differences with *P *< 0.05. The ratios for all peptides corresponding to amino acids 20-35 of fibrinogen alpha are shown in Additional file 1, Figure S2.

### Synovial fluid contains multiply modified fibrinopeptide A

Interestingly, fibrinopeptide A also contains a known phosphorylation site on fibrinogen alpha (S22) [19], which is most likely generated by a member of the casein kinase II (CK2) family [20]. To determine if the presence or absence of phosphorylation was in any way linked to the citrullination of R35, we extended our database search to include this possibility and were able to identify two peptides (with sequences ADpSGEGDFLAEGGGVcR and DpSGEGDFLAEGGGVcR) containing both a phosphorylated S22 and a citrullinated R35 in a number of the patients (Additional file 1, Figure S3A). We then also determined the XIC areas of these peptides in all patients, showing that the peptides containing both modifications showed a similar behavior to the peptides containing only the citrullination site (for example, more present in RA patients), whereas peptides containing only the phosphorylation site were equally abundant in all patients (Additional file 1, Figure S3B). A final fibrinopeptide A-derived phosphopeptide containing a citrulline residue with sequence pSGEGDFLAEGGGVcR was identified with low identification scores in some of the samples, but was found to be present in only very low amounts and was, therefore, not included in this analysis (data not shown).

## Discussion

Many citrullinated proteins have been identified in the inflamed joints of RA patients and a number of these have been shown react *in vitro *with ACPA. A prominent candidate to be the main target for ACPA is fibrin(ogen), which is known to be present in large amounts in inflamed joints and which is known to be present in a citrullinated form in the synovial fluid of RA patients [3,21]. ACPA can indeed bind to citrullinated fibrinogen, in particular the alpha chain. Recently, it was shown that in inflamed joints also significant amounts of peptides can be found that are derived from fibrinogen, due to endogenous proteolytic activity [15]. Here, we showed that citrullinated versions of these peptides can be found in the synovial fluid of inflamed joints. While some of these citrullinated peptides were equally abundant in the RA patients and the control group with patients suffering from other inflammatory joint diseases, others appeared to be more abundant in RA patients (Table 1). Consistent with that observation, PAD activity in joints is known to be elevated in such other inflammatory diseases as well. The higher level of some of the peptides in RA patients suggests that a different immune response toward these peptides might occur in RA patients, leading to, for example, prolonged retention of these peptides, which, in turn, could contribute to increased immune system activation. Although there was significant diversity in the levels of citrullinated peptides in the control group, with especially patient S2 (ankylosing spondylitis) showing very high levels, no obvious relation with their diagnosed disease was apparent (Additional file 1, Table S1). This diversity among the patients in the control group confirms that the citrullinated peptides arose from inflammatory events that can also occur in diseases other than RA, which is not unexpected as many rheumatic diseases show overlap in the symptoms observed. An ideal control material would be synovial fluid from healthy individuals, but for obvious reasons this material is less readily available, not only due to ethical issues, but also because the amount of synovial fluid in noninflamed joints is much smaller.

The citrullinated peptides we found to be most abundantly present in RA patients covered mainly two distinct regions in the alpha chain of fibrinogen, amino acids 20-35 and 414-433. These regions have been shown to be susceptible to citrullination in the past and it was shown that the arginine residues in these sequences (R35, R425 and R426) can be citrullinated by both the PAD2 as well as the PAD4 enzymes [22], both known to be present in inflammatory cells in the synovium [23]. Together, these PAD enzymes are known to be able to citrullinate two-thirds of all arginine residues in fibrinogen [22], but little is known about which citrullinated residues contribute most to eliciting the anti-citrullinated protein immune response. The fact that specific citrullinated fibrinogen-derived peptides reside in the synovial fluid makes them prime candidates for having a role in the immune response against citrullinated epitopes.

The fibrinogen region between amino acids 414-433 is known to be autoantigenic in RA patients [6,22], but for the citrullinated fibrinopeptide A (amino acids 20-35) no data on autoantigenicity is available, most likely because it is not part of the mature fibrinogen protein that has been used in such studies most frequently.

Interestingly, the fact that fibrinopeptide A, after cleavage from the fibrinogen precursor, contains a C-terminal arginine residue makes it an excellent substrate for PAD enzymes, which are able to efficiently convert such residues to citrulline (data not shown).

Both sequence elements were represented by a variety of peptides, differing in either their N- or C-terminal sequence (Figures 4 and S1), most likely due to *in vivo *proteolytic activities. While it is difficult to predict the enzyme responsible for their generation, proteases abundantly present in inflammatory cells, such as matrix metalloproteinases, elastases or caspases are likely involved. The two cleavage sites generating the full amino acids 20-35 fragment, however, are obvious, as this corresponds exactly to the borders of fibrinopeptide A, which is released from the A-alpha chain by cleavage at the N-terminal side of amino acid 20 by the signal peptidase and at the C-terminal side of amino acid 35 by thrombin [7,24] The latter cleavage is one of the first steps of the clotting cascade and therefore the presence of (derivatives of) fibrinopeptide A is not very surprising, although its presence in a citrullinated fashion has not been reported before. Because thrombin cleavage at that position is dependent on the presence of an arginine residue, the citrullination of the C-terminal arginine of fibrinopeptide A has most likely occurred after thrombin cleavage. Citrullination of R35 in the uncleaved fibrinogen protein would inhibit thrombin cleavage and might, therefore, have implications on the efficiency of clotting in inflamed joints [25]. The cleavage generating the N-terminus of the amino acids 414-433 fragment is most likely mediated by cathepsin D, which can cleave fibrinogen at that position in the process of clearing fibrin cloths [26].

Fibrinogen is certainly not the only citrullinated antigen present in synovial fluid as, for example, citrullinated fibronectin and vimentin have also been found [21]. However, the presence of high levels of endogenous citrullinated peptides derived from the protein is quite unique to fibrinogen, and could potentially provide a direct link between with the generation of an immune response targeting citrullinated epitopes. From a diagnostic point of view, such peptides might be very interesting to discriminate RA from closely related inflammatory diseases that also affect the joints. This could be achieved either by employing targeted mass spectrometric techniques or by the development of alternative, for example, antibody-based methods that would allow the specific detection of these citrullinated peptides in synovial fluid samples.

## Conclusions

We have shown the presence of increased amounts of specific endogenous citrullinated peptides, derived from fibrinogen, in the synovial fluid of RA patients. Citrullinated fibrinopeptide A was found to be present more abundantly in RA patients compared to control patients and compared to the not citrullinated fibrinopeptide. This elevated presence could indicate a role for such endogenously generated, citrullinated peptides in the development of the anti-citrullinated protein antibody (ACPA) immune response that is often seen in RA patients.

## Abbreviations

ACN: acetonitrile; ACPA: anti-citrullinated protein antibodies; CCP: cyclic citrullinated peptide; ELISA: enzyme-linked immunosorbent assay; FA: formic acid; HLA: human leukocyte antigen; IFN: interferon; IL: interleukin; kDa: kiloDaltons; LC-MS/MS: liquid chromatography-tandem mass spectrometry; MHC: major histocompatibility complex; MS: mass spectrometry; m/z: mass-to-charge ratio; PAD: peptidylarginine deiminase; RA: rheumatoid arthritis; RF: rheumatoid factor; SF: synovial fluid; TNF: tumor necrosis factor; XIC: extracted ion chromatogram.

## Competing interests

The authors declare that they have no competing interests.

## Authors' contributions

RR, MEA and NFCV performed the proteomics experiments. RR and MEA conducted the proteomic data analyses. BB and JJBCvB provided and characterized samples. RR, GJMP and AJRH designed and supervised the research. RR, JJBCvB, MEA, BB, GJMP and AJRH wrote the paper. All authors read and approved the final manuscript.

## Supplementary Material

Additional file 1 **A PDF file containing all supplementary methods, tables and figures as referred to in the main text**. The additional file contains supplementary methods on 'LC-MS/MS' and 'Identification and quantitation of endogenous peptides', a link to all identification results as deposited in the ProteomeCommons.org Tranche, supplementary tables S1 ('List of patients included and CCP status of RA patients') and S2 ('LC-MS characteristics of identified citrullinated peptides and their noncitrullinated counterparts') and supplementary figures S1 ('Abundance of fibrinogen amino acids 414-433-derived peptides in RA patients and controls'), S2 ('Relative abundance of citrullinated peptides and their noncitrullinated counterparts') and S3 ('The presence of phosphorylated and citrullinated peptides in synovial fluid'). Click here for file
