# Supplementary material for: Elevated levels of fibrinogen-derived endogenous citrullinated peptides in synovial fluid of rheumatoid arthritis patients
Source: Arthritis Res Ther. 2012 May 14;14(3):R114. doi: 10.1186/ar3840 (PMC3446491; doi:10.1186/ar3840)
Supplement: Additional file 1 — A PDF file containing all supplementary methods, tables and figures as referred to in the main text. The additional file contains supplementary methods on 'LC-MS/MS' and 'Identification and quantitation of endogenous peptides', a link to all identification results as deposited in the ProteomeCommons.org Tranche, supplementary tables S1 ('List of patients included and CCP status of RA patients') and S2 ('LC-MS characteristics of identified citrullinated peptides and their noncitrullinated counterparts') and supplementary figures S1 ('Abundance of fibrinogen amino acids 414-433-derived peptides in RA patients and controls'), S2 ('Relative abundance of citrullinated peptides and their noncitrullinated counterparts') and S3 ('The presence of phosphorylated and citrullinated peptides in synovial fluid'). [file ar3840-S1.PDF]

Elevated levels of fibrinogen-derived endogenous citrullinated peptides in synovial fluid of rheumatoid arthritis patients

Reinout Raijmakers[1], Joyce J.B.C. van Beers[2], Mahmoud El-Azzouny[1], Natasja F.C. Visser[1], Borut Božič[3], Ger J.M. Pruijn[2] and Albert J.R. Heck[1]

[1] Biomolecular Mass Spectrometry and Proteomics, Bijvoet Center for Biomolecular Research and Utrecht Institute for Pharmaceutical Sciences, Utrecht University and Netherlands Proteomics Centre, Padualaan 8, Utrecht, 3584 CH, The Netherlands

[2] Department of Biomolecular Chemistry, Nijmegen Center for Molecular Life Sciences, Institute for Molecules and Materials, Radboud University Nijmegen, Geert Grooteplein 26, Nijmegen, 6525 GA, The Netherlands

[3] University Medical Centre Ljubljana, Department of Rheumatology, Immunology Laboratory, Vodnikova 62, Ljubljana, 1000, Slovenia

Correspondence:

Reinout Raijmakers and Albert J.R. Heck

Padualaan 8

3584 CH Utrecht

R.Raijmakers@uu.nl and A.J.R.Heck@uu.nl

SUPPLEMENTARY METHODS

LC-MS/MS

Peptides were injected onto a nano-scale liquid chromatography system. An Agilent 1100 series LC system was equipped with an 20 mm Aqua C18 (Phenomenex, Torrance, CA) trapping column (packed in-house, i.d., 100 µm; resin, 5 µm) and a 250 mm ReproSil-Pur C18-AQ analytical column (packed in-house, i.d., 50 µm; resin, 3 µm). Trapping was performed at 5 µL/min for 10 min and elution was achieved with a gradient of 0-32% B in 60 min, 32-40% B in 5 min, 40-100% B in 2 min and 100% B for 2 min leading to a total analysis time of 90 minutes. The flow rate was passively split from 0.4 ml/min to 100 nl/min when performing the elution analysis. Nanospray was achieved using a distally coated fused silica emitter (New Objective, Cambridge, MA) (o.d., 360 µm; i.d., 20 µm, tip i.d. 10 µm) biased to 1.8 kV. The LC system was coupled to a 7 Tesla LTQ-FTICR mass spectrometer (Thermo Scientific, Bremen, Germany). The mass spectrometer was operated in the data-dependent mode to automatically switch between MS and MS/MS acquisition. Survey full scan MS spectra were acquired from m/z 350 to m/z 1500 in the FTICR with a resolution of R=100,000 at m/z 400 after accumulation to a target value of 1,000,000. The five most intense ions were fragmented in the linear ion trap using collisionally induced dissociation at a target value of 30,000.

IDENTIFICATION AND QUANTITATION OF ENDOGENOUS PEPTIDES

Spectra were processed with Bioworks 3.3 (Thermo, Bremen, Germany) and the subsequent data analysis was carried out using the Mascot (version 2.1.0) software platform (Matrix Science, London, UK) against all human proteins in the SwissProt database (version 56.2). The search criteria included methionine oxidation, arginine citrullination and asparagine and glutamine deamidation as variable modifications. The peptide tolerance was set to 5 ppm and the MS/MS tolerance to 0.6 Da. All searches were done without enzyme restrictions and peptides were, unless described differently, identified with a minimum Mascot score of 35 and a p value of 0.05, leading to a false discovery rate of <2% as estimated by decoy database searching. For spectral counting, peptide spectrum matches with a Mascot score of at least 20 were included. Quantitation of peptide intensities was done based on extracted ion chromatogram (XIC) surface areas, calculated using XCalibur. Finally, all peptide intensities were normalized for the average intensities of the spiked internal standard peptides. Annotation of MS/MS spectra was done using Scaffold (Proteome Software). All identification results are available for download from the ProteomeCommons.org Tranche, the details of which are supplied in the supplementary results.

All identification results are available for download in a Scaffold file that may be downloaded using the following hash from ProteomeCommons.org Tranche (https://proteomecommons.org/tranche/):

RX0IqP0a+AzZgGQb3YYhEA/0asuEN6xkYuREPyDTE9Vc+bi9UJd+gi/3Zg6JxadcKWe
vgF5aWwT/+L6MeC1XxOcF3xUAAAAAAAADiw==

The viewer is downloadable from http://www.proteomesoftware.com.

SUPPLEMENTARY TABLES

Table S1. List of patients included and CCP status of RA patients.

Code	Diagnosis	CCP2
**S2**	ankylosing spondylitis	n.d.
**S3**	gout	n.d.
**S4**	osteoarthritis	n.d.
**S13**	rheumatoid arthritis	+
**S20**	ochronosis	n.d.
**S22**	rheumatoid arthritis	-
**S25**	rheumatoid arthritis	+
**S28**	rheumatoid arthritis	+
**S29**	rheumatoid arthritis	+
**S32**	psoriatic arthritis	n.d.
**S33**	psoriatic arthritis	n.d.
**S34**	rheumatoid arthritis	+
**S37**	psoriatic arthritis	n.d.
**S40**	ankylosing spondylitis	n.d.
**S42**	rheumatoid arthritis	+
**S44**	gout	n.d.
**S45**	rheumatoid arthritis	-
**S47**	rheumatoid arthritis	-
**S49**	rheumatoid arthritis	+
**S51**	psoriatic arthritis	n.d.
**S53**	rheumatoid arthritis	+

n.d.: not determined

Table S2. LC-MS characteristics of identified citrullinated peptides and their non-citrullinated counterparts.

	Main charge state		Observed m/z		Observed RT	
**Sequence**	**R**	**Cit**	**R**	**Cit**	**R**	**Cit**
**DSGEGDFLAEGGGVcR**	2	2	733.33	733.83	53.18	61.08
**SGEGDFLAEGGGVcR**	2	2	675.82	676.31	49.9	57.73
**EGDFLAEGGGVcR**	2	2	603.79	604.28	48.12	56.80
**FLAEGGGVcR**	2	2	453.25	453.74	35.66	44.53
**EEVSGNVSPGTRcR**	3	2	463.24	694.84	24.83	27.25
**EEVSGNVSPGTRcRE**	3	2	506.25	759.36	25.27	27.89
**EEVSGNVSPGTcRREY**	3	2	560.60	840.90	29.80	34.61
**EEVSGNVSPGTRcREY**	3	2	560.60	840.90	29.80	34.61
**ELEcRPGGNEITRGGSTSY**	2	2	641.65	641.97	36.89	39.36
**NcRGDSTFESKSY**	3	2	464.21	696.31	29.37	34.75
**DEAGSEADHEGTHSTKcRGHA**	3	2	523.73	698.3	n.d.	n.d.
**GSTGNcRNPGSSGTGGTATWKPGSSGP**	2	2	792.37	792.70	n.d.	n.d.
**SETEScRGSESGIFTNTK**	2	2	610.62	610.95	33.65	35.36
**SSSYSKQFTSSTSYNcRGDSTF**	4	3	733.58	978.09	41.86	46.35
**TcRGGSTSYGTGSETESPRNPS**	2	2	709.99	710.32	25.88	35.86
**TSEASSSSSSSSSSRScRScRSL**	n.d.	2	n.d.	1105.00	n.d.	n.d.
**GLADNTNDLEKcRcR**	n.d.	3	n.d.	501.92	n.d.	n.d.

n.d.: not determined (either non-citrullinated peptide not detected or ambiguous RT)

SUPPLEMENTARY FIGURES

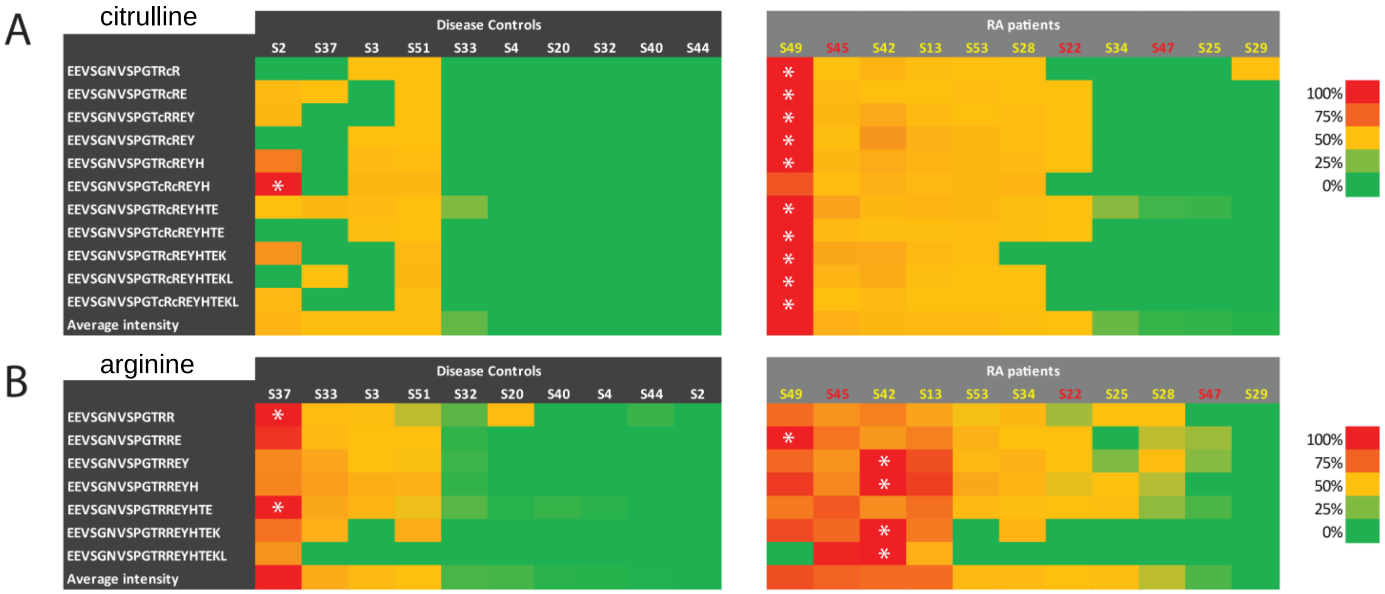

Figure S1. Abundance of fibrinogen aa 414-433 derived peptides in RA patients and controls. Heatmap displaying the relative intensity (as determined from the extracted ion chromatograms, XIC) of citrullinated peptides (panel A) and their non-citrullinated counterparts (panel B) in RA patients and control patients. Color coding is applied per peptide sequence with the patient containing the highest amount of peptide colored in red, the lowest colored in green and the other patients colored according to the color scale shown on the right of the panel. White asterisks indicate in which patient the highest abundance of a specific peptide was observed. For the RA patients, the code of CCP2+ patients is shown in yellow and of CCP2- patients in red. Patients are sorted according to the average intensity of all listed peptides, shown at the bottom of each panel.

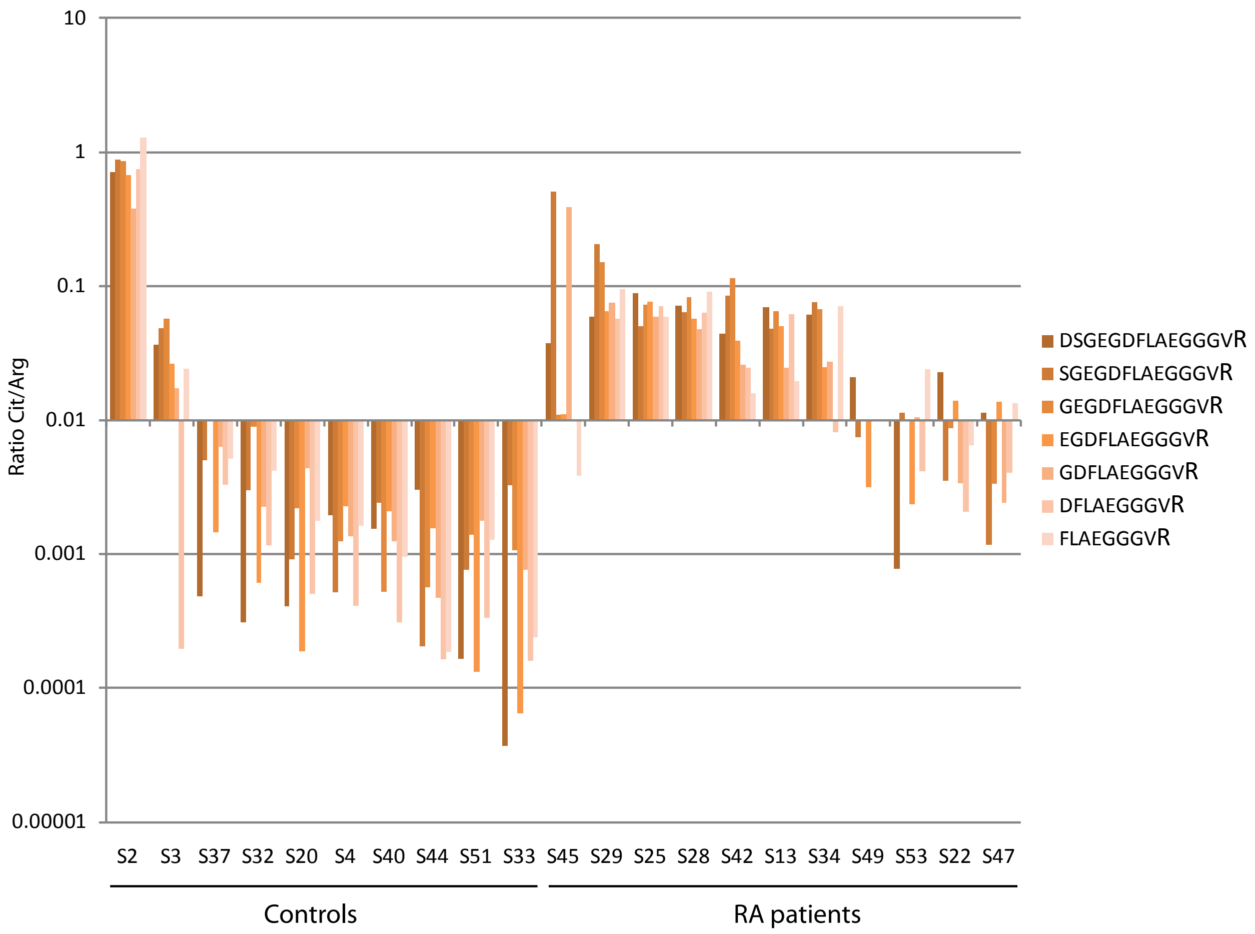

Figure S2. Relative abundance of citrullinated peptides and their non-citrullinated counterparts. Bar graph showing the ratio between the intensity of a all identified fibrinopeptide A derived citrullinated peptides versus their non-citrullinated variant in RA patients and control patients on a base 10 logarithmic scale. When no signal for the peptide was detected, an intensity value equivalent to the background signal was assigned for this calculation. If both the signal of the citrullinated and the non-citrullinated peptide were missing, the ratio is omitted. Patients are sorted per group according to the average of all observed ratios.

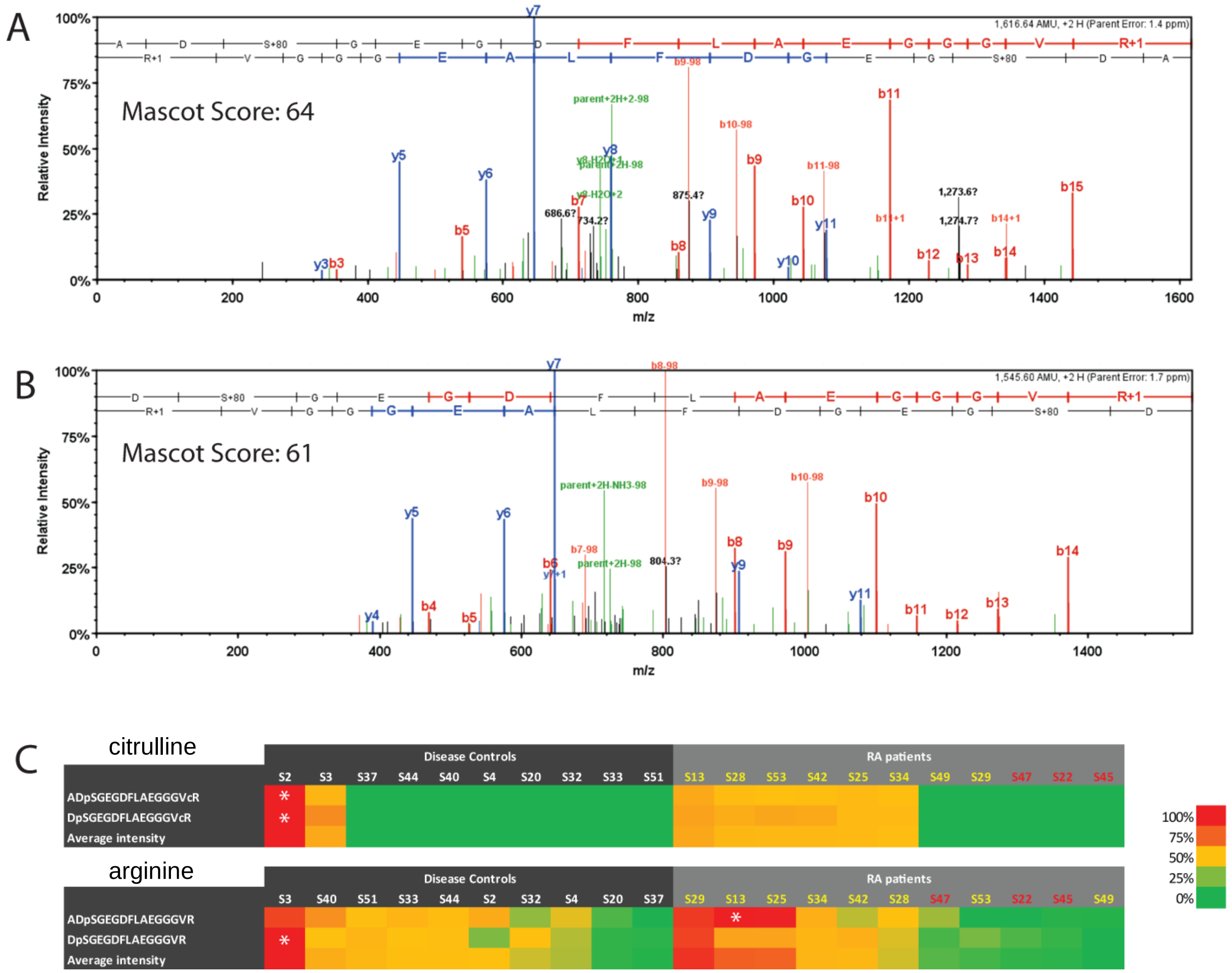

Figure S3. The presence of phosphorylated and citrullinated peptides in synovial fluid. Panels A and B show tandem MS spectra of peptides ADpSGEGDFLAEGGGVcR and DpSGEGDFLAEGGGVcR, respectively, containing both a phosphorylated serine 22 at as well as a citrullinated arginine 35. Fragment ions used for the identification are colored blue if they contain the N-terminus of the peptide (y-ions), red if they contain the C-terminus of the peptide (b-ions) and green for all other fragment ions. Fragments showing a "neutral loss" of the phosphate group, and thus confirming the presence of a phosphorylated residue are marked with "-98". The corresponding identification scores (Mascot scores) are shown in each of the spectra. Panel C shows the heatmap of the XIC intensities of the identified S22 phosphorylated alpha fibrinogen peptides in RA and control patients, with (top panel) and without (bottom panel) citrullination at position 35. Color coding is applied per peptide sequence with the patient containing the highest amount of peptide colored in red, the lowest colored in green and the other patients colored according to the color scale shown on the right of the panel. White asterisks indicate in which patient the highest abundance of a specific peptide was observed. For the RA patients, the code of CCP2+ patients is shown in yellow and of CCP2- patients in red. Patients are sorted according to the average intensity of all listed peptides, shown at the bottom of each panel.
